# Revealing the Mutual Information between Body-Worn Sensors and Metabolic Cost in Running

**DOI:** 10.3390/s23041756

**Published:** 2023-02-04

**Authors:** Tobias Baumgartner, Stefanie Klatt, Lars Donath

**Affiliations:** 1 Institute of Exercise Training and Sport Informatics, Department of Cognitive and Team/Racket Sport Research, German Sport University Cologne, 50933 Cologne, Germany; 2 School of Sport and Health Sciences, University of Brighton, Eastbourne BN20 7SR, UK; 3 Department of Intervention Research in Exercise Training, German Sport University Cologne, 50933 Cologne, Germany

**Keywords:** running power, running economy, accelerometers, bluetooth low energy beacons, convolutional neural networks, information bottleneck

## Abstract

Running power is a popular measure to gauge objective intensity. It has recently been shown, though, that foot-worn sensors alone cannot reflect variations in the exerted energy that stems from changes in the running economy. In order to support long-term improvement in running, these changes need to be taken into account. We propose leveraging the presence of two additional sensors worn by the most ambitious recreational runners for improved measurement: a watch and a heart rate chest strap. Using these accelerometers, which are already present and distributed over the athlete’s body, carries more information about metabolic demand than a single foot-worn sensor. In this work, we demonstrate the mutual information between acceleration data and the metabolic demand of running by leveraging the information bottleneck of a constrained convolutional neural network. We perform lab measurements on 29 ambitious recreational runners (age = 28 ± 7 years, weekly running distance = 50 ± 25 km, $\dot{V}O_{2\max}$ = 60.3 ± 7.4 mL · min^−1^·kg^−1^). We show that information about the metabolic demand of running is contained in kinetic data. Additionally, we prove that the combination of three sensors (foot, torso, and lower arm) carries significantly more information than a single foot-worn sensor. We advocate for the development of running power systems that incorporate the sensors in watches and chest straps to improve the validity of running power and, thereby, long-term training planning.

## 1. Introduction

Accurate measurement of metabolic demand during running is relevant for both athletic training and rehabilitation [1]. Gold-standard methods to measure metabolic demand (such as indirect calorimetry via respiratory gas exchange) are infeasible in the field and unpractical for everyday use by recreational runners. Similar to the use of power meters in cycling, foot sensors have recently been used for measuring power when running [2,3,4,5,6]. In contrast to cycling though [7], the efficiency with which an individual converts metabolic intake into the force exerted on the ground during running, known as the running economy (RE) [8], can vary widely between individuals [9]. As such, it is important for long-term training success to consider changes in the running economy [10]. It has recently been shown that foot-worn sensors are limited when it comes to adaptation to changing the running economies [11].

In this work, we explore the possibility of improving the measurement of metabolic demand during running by leveraging additional sensors commonly worn by recreational runners [12]. By collecting data from a combination of foot-, torso-, and arm-based sensors, we aim to gain a more comprehensive understanding of the kinetics of an athlete and the relationship between kinetics, kinematics, and metabolism. Our results show that the combination of these three sensors provides additional information about an athlete’s metabolism. In addition, this investigation motivates further studies into the link between kinetics, kinematics, and metabolism and the potential for using these insights to inform the development of optimal running form.

Long-distance running performance can be assessed by energy expenditure. Humans have limited, immediately available energy storage [13,14,15], and, therefore, the rate of energy consumption at a certain pace is the limiting factor for running performance [16]. A good proxy for energy expenditure is oxygen uptake, which has been shown to correlate highly with energy expenditure [17,18]. In our experiments, we directly measure the consumed oxygen $\dot{V}O_{2}$ via a spirometry system, measuring the rate of oxygen consumption in liters per minute ($L\cdot \min^{-1}$). The factor between mechanical and metabolic oxygen consumption, i.e., the relative difference between the external work performed and energy consumed, is defined as the running economy RE [8,19]. The more energy that is lost without being used for forward propulsion, the lower the RE [20]. The running economy can also be described as the factor between external and internal work, and we distinguish between the external mechanical energy that is observed in kinetic forces and the internal metabolic energy that is consumed by the athlete [20,21].

Our work is based on a study by Baumgartner et al. [11]. In their study, the authors aimed to determine the validity of foot-worn sensors for measuring running power. They found that while these sensors can accurately measure power under steady RE conditions, they are no longer valid when RE is altered. In their study, they conducted an experiment to alter participants’ running style in ways that are known to affect RE [22,23] and found that the changes in metabolic cost were not reflected in power measured by the foot-worn sensors. They concluded that running power measured by these sensors is only a useful measure in situations where the athlete’s running style does not vary or change. Therefore, using these devices to organize long-term training may not have the intended effects, as changes in RE can lead to arbitrary changes in displayed power. In this work, we showcase unpublished data that was recorded simultaneously with the previous work by Baumgartner et al. [11].

The sensors in this previous investigation were based on acceleration data collected at the foot. In this follow-up investigation, we show that the previous results could be improved by using two additional sensors that many recreational runners already wear for their routine running: a watch on the arm and a chest strap. Using sensors at three different locations could yield a more accurate method for measuring running power. By measuring acceleration at three different body positions, it is possible to capture more information about the movement of the body on top of the forces being applied to the ground. This additional information can be used to accurately calculate mechanical power, which more closely relates to metabolic power than the power measured by just a single foot-worn sensor.

In our experiment, we train deep neural networks to demonstrate the connection between data from accelerometers and respiratory gases. During the training process, the network is presented with a set of input-output pairs and attempts to learn a function that maps the input to the output. The network makes predictions for a given input and compares these predictions to the true output. The difference between the predicted and true output is known as the error. The network consists of multiple layers for transforming its input into an output. These transformations consist of multiplications and additions of various combinations of inputs, as well as pooling operations (such as averaging or taking the maximum) and non-linearities (setting any value below zero to zero). Each layer has a certain set of parameters that define its transformation and, therefore, determine how some input is transformed into an output [24]. During training, the network adjusts its parameters in an attempt to minimize the error. The process of adjusting the parameters is known as backpropagation: At each layer, the error is used to calculate the gradient of the error with respect to the layer’s parameters. The gradient is then used to update the parameters in a direction that reduces the error.

In our proof below, we use arguments inspired by the information bottleneck method as proposed by Tishby et al. [25]. They propose a framework for understanding the trade-off between the amount of information that can be preserved in a signal and the amount of information that can be transmitted through a communication channel. In our scenario, we use a channel with known, limited capacity, which is purposefully underpowered. We achieve this by defining a model with less trainable parameters than data points. Thereby, we know that the only way that our model can pass information through this channel is by encoding signals about the actually observed phenomenon. We use this bottleneck to demonstrate that there is, in fact, information about the metabolic energy expenditure contained in accelerometer data. Moreover, using sensors at three separate, distributed locations allows for capturing more of that contained information. It follows that novel products for running power should combine foot-worn sensors with sensors in watches and heart-rate chest straps.

The objective of the present study is to investigate avenues for improving the measurement of metabolic demand in the field. We aim to mitigate the shortcomings of current commercial products by incorporating additional sensors typically worn by recreational runners. While a single foot-worn sensor does not contain enough information about the metabolic demand under changing running economy, we hypothesize that three sensors on the foot, torso, and arm allow improving approximations.

## 2. Materials and Methods

### 2.1. Subjects and Testing Procedure

A total of 32 moderately endurance-trained runners participated in a controlled crossover trial. Due to partial Bluetooth connection problems, three out of the 32 subjects had to be excluded from this investigation, and in the following, we use data from the remaining 29 subjects: 19 males and 10 females with a mean age of 28 ± 7 years and a mean BMI of 21.6 ± 1.6. These participants had run an average of 50 ± 25 km per week in the preceding four weeks, had a mean running $\dot{V}O_{2\max}$ of 60.3 ± 7.4 mL · min^−1^·kg^−1^, had at least one year of distance running experience, and were injury-free for at least three months. Participants wore their own footwear and participated in a combined incremental and ramp exercise test to determine their velocity at the aerobic threshold (AeT) [26] and their $\dot{V}O_{2\max}$ [27]. The test protocol, which took place in a single laboratory visit, included an incremental $\dot{V}O_{2\max}$ signal sill test followed by a 25-min treadmill run with varying predetermined spatiotemporal running parameters after a 30-min rest.

**Figure 1:**
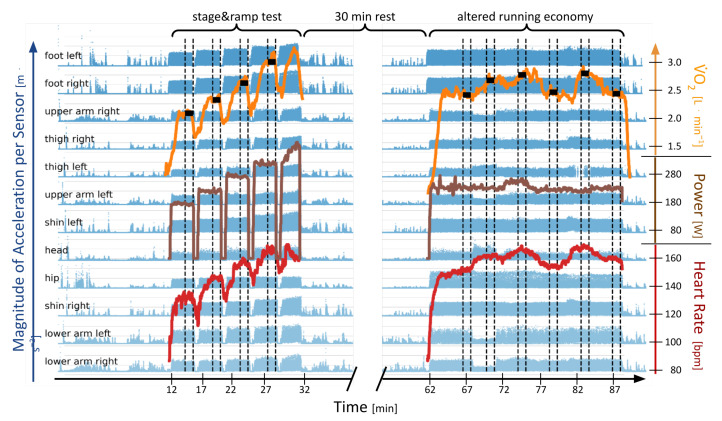
Overview of all collected data for a single athlete: the magnitude of accelerometers at 12 positions on the body (blue); red: heart rate; brown: running power (Stryd); orange: $\dot{V}O_{2}$; dashed lines = time section used for tests; in the left half is the last 60 s of each stage in the graded exercise test; on the right are the last 60 s of each modified running condition; black boxes mark the $\dot{V}O_{2}$ average for each section. Best viewed in color.

**Figure 2:**
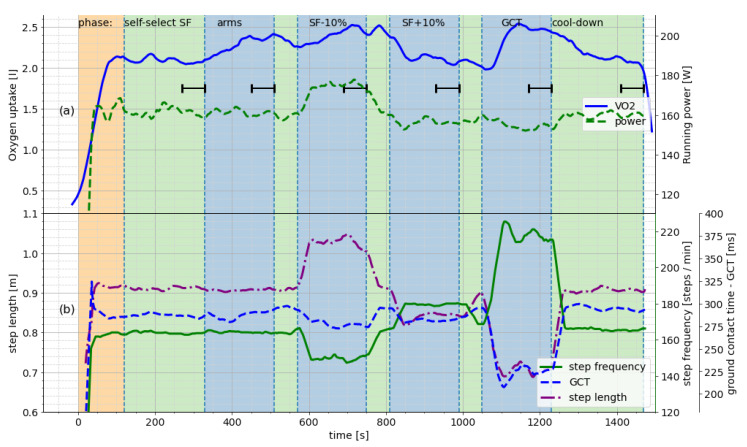
Exemplar protocol for the second part of the experiment and varying spatiotemporal parameters for a single subject. (**a**) Oxygen consumption $\dot{V}O_{2}$ and running power as measured using a single foot-worn commercial sensor during the running conditions. (**b**) Spatiotemporal running parameters of the subject in reaction to instructions for the experimental conditions. Reprinted with permission from Baumgartner et al. [11]. Best viewed in color.

During the incremental exercise test, subjects performed four 3 min stages at increasing speeds, of which we considered the last 60 s each, as indicated by the dashed lines in Figure 1. During the “altered running economy” portion of the experiments, athletes ran with (1) their own running form, (2) increased step frequency, (3) decreased step frequency, (4) shortened ground contact time, (5) immobilized arms, and (6) their own form again to gauge fatigue. Each condition was run for 3 min, with a 1 min washout phase at a normal running form in between. The last 60 s of each condition were used for our investigation. We removed data from condition (5) from this investigation since it is not a natural running form and randomized the order of the remaining altered conditions. Figure 2 illustrates the testing procedure for the altered running conditions that are part of the experiment.

The participants were instructed to refrain from intense exercise for 48 h prior to the test. Throughout the above tests, participants wore a heart rate monitor chest strap (Garmin, Olathe, KS, USA). Spirometric data was collected using a breath-by-breath spirometric system (Zan 600, Zan Messgeräte, Oberthulba, Germany) and calibrated prior to each test, following the manufacturer’s instructions. In addition, capillary blood samples from the earlobe were taken during the 30 s rest periods between stages in the ramp test for lactate analysis (EBIOplus; EKF Diagnostic Sales, Magdeburg, Germany). The lactate analysis informed the pace at which the test with the modulated running economy was performed. All data, their synchronization, and the order of experiments are illustrated in Figure 1.

### 2.2. Accelerometer Data

**Figure 3:**
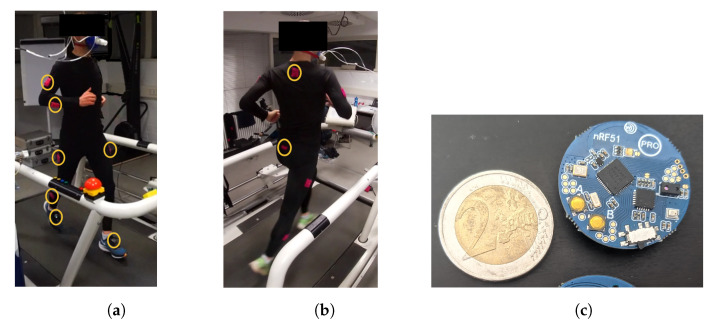
(**a**,**b**) Picture of sensor suit from front/back. Sensor Beacons are attached to the runner in pink sewn-on pockets (yellow circles) at 12 locations: 2 × feet, 2 × shin, 2 × thigh, 2 × upper arm, 2 × lower arm, hip, and neck. (**c**) Picture of the used Bluetooth beacon (weight = 6 g, diameter = 3 cm, coin for scale).

Subjects wore custom-made sensor suits during the study, which consisted of compression pants and long-sleeve shirts with pockets that held battery-powered microcontroller boards (Bluetooth beacons). To ensure a secure fit and minimize sensor wobbling, each subject received a garment in their size out of a choice of five typical clothing sizes (cf. Figure 3a,b).

The Bluetooth beacons used in this study were about the diameter (3 cm) and weight (6 g) of a 2 Euro coin (cf. Figure 3c). They were based on the nRF51822 System-on-Chip microcontroller (Nordic Semiconductors, Trondheim, Norwegen) with built-in Bluetooth Low Energy and contained an MPU-6050 accelerometer (Invensense, San Jose, CA, USA) and other minor peripherals, as well as input and output capabilities. These beacons were powered by rechargeable 3.7 V cell batteries (LIR2032H) and were ordered pre-assembled from AliExpress in 2021 (search terms: “NRF51822 Bluetooth beacon”).

A huge advantage of Bluetooth low-energy (BLE) is the namesake low energy consumption, which allowed us to run the described configuration for roughly four hours off batteries and without cables. This advantage was bought with low throughput. We developed customized firmware that allowed us to compress and bundle multiple sensor readings and subsequently unpack them onto the receiving master device. Using this method, we achieved accelerometer readings of up to 60 Hz.

We calibrated the sensors by laying them flat on the table for 30 s. Then subjects stood still with their arms down and jumped from just their ankles (i.e., trying to jump without bending their knees), facing the front of the treadmill. This way, we got the relative orientation of the accelerometer module of the sensors to the world-coordinate system of the treadmill laboratory.

Before starting the tests, the subjects performed multiple jumps in order to later synchronize the data with video recordings. The frame in which the athlete reaches the highest point of their jump corresponds to the lowest acceleration between two peak accelerations of the jump-off and landing frames. An additional beacon with an extra button was also built for the purpose of marking the exact time at which the spirometric recording began, ensuring that $\dot{V}O_{2}$ and accelerometer data could be synchronized.

We recorded data from a total of 12 locations throughout the body: 2 × foot, 2 × shin, 2 × thigh, 2 × upper arm, 2 × lower arm, hip, and neck (cf. Figure 3a,b). We will later focus on the output of just three sensors for each subject in order to create a realistic scenario in which recreational runners wear foot sensors, heart rate monitors, and watches.

### 2.3. Relation between Energy and Sensors

Through our experiment, we show that there is information about the metabolism contained in acceleration data. We show the mutual information between the consumed oxygen $\dot{V}O_{2}$ and the measured acceleration A. In and of itself, this connection is fairly straightforward, acceleration at the foot during running can contain information about metabolism because the forces exerted on the ground during running can be related to energy expenditure. Force is applied to the ground through the feet, which causes acceleration. This acceleration can be measured using accelerometers, and the magnitude of the acceleration carries information about the energy expenditure during running. The energy expenditure can, in turn, be related to the person’s metabolism, as the body must use energy from metabolism to produce the force needed to run. Therefore, by measuring acceleration at the foot during running, it is possible to indirectly obtain information about the person’s metabolism.

**Figure 4:**
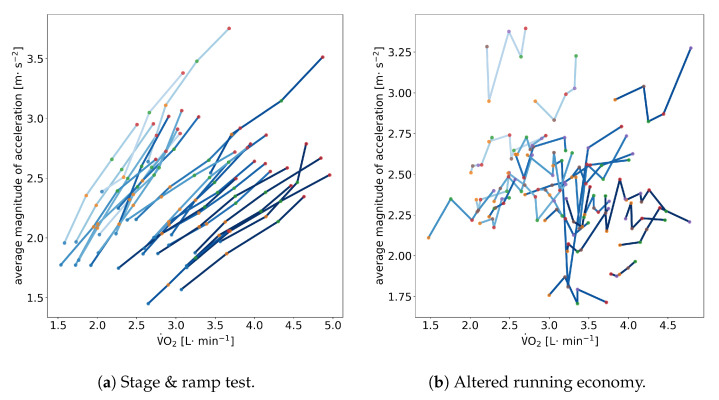
Correlation between $\dot{V}O_{2}$ and the average magnitude of acceleration A during (**a**) the initial stage-ramp test and during (**b**) the running experiment with altered running economy. Each line signifies a single athlete. The shade of blue of the line signifies the body weight of the athlete (darker = heavier). Dots signify the different stages in (**a**) or altered running conditions in (**b**).

Previous work has shown that there is not necessarily a strict correlation between $\dot{V}O_{2}$ and running power as measured by a single acceleration sensor at the foot [11]. We illustrate this same observation in Figure 4, in which we show correlation plots between the consumed oxygen $\dot{V}O_{2}$ and the average magnitude of acceleration of a single foot-worn sensor. While there is a linear dependency for each athlete between magnitude of acceleration and $\dot{V}O_{2}$ during the stage & ramp test (cf. Figure 4a), this correlation break when altering the spatiotemporal running parameters (cf. Figure 4b).

When the running economy is changed, the factor between mechanical and metabolic energy expenditure changes, the acceleration is a proxy for mechanical force, and once RE changes over the course of a longer training period, the connection between mechanical and metabolic energy expenditure is broken.

In our experiment, we measured acceleration A and consumed oxygen $\dot{V}O_{2}$. A was a proxy for the force, and thereby mechanical energy $E_{\mathrm{mech}}$, that is exerted into the ground via $F_{\mathrm{GRF}}=m\cdot A$. $\dot{V}O_{2}$ is proportional to the consumed energy of the athlete $E_{\mathrm{metab}}$. RE incorporates all differences between $E_{\mathrm{metab}}$ and $E_{\mathrm{mech}}$ that stem from the individual running form. The running economy (RE) also accounts for energy lost as heat due to friction and other inefficiencies that are inherent to the human body when transforming foodstuff into motion.

Overall we consider the above terms in the following relationship to each other:

(1) $$\begin{array}{ccc} \dot{V}O_{2}\propto E_{\mathrm{metab}} & =E_{\mathrm{mech}} & +E_{\mathrm{lost}}\\ \; & =\underset{=f(F_{\mathrm{GRF}},\theta )}{\underset{︸}{E_{\mathrm{ground}}}}\; & +\underset{RE}{\underset{︸}{E_{\mathrm{remain}}+E_{\mathrm{lost}}}} \end{array}$$

$\dot{V}O_{2}$ is proportional to $E_{\mathrm{metab}}$. $E_{\mathrm{metab}}$ equals the externally measurable $E_{\mathrm{mech}}$ plus the lost energy $E_{\mathrm{lost}}$ (which is partially described by RE). $E_{\mathrm{mech}}$ is always smaller than $E_{\mathrm{metab}}$. $E_{\mathrm{mech}}$ can be expressed as a function of all forces F which are exerted by the body. The energy expended into the ground ($E_{\mathrm{ground}}$) plus energy expended for arm (and other variations of) motion ($E_{\mathrm{remain}}$) account for the measurable difference between $E_{\mathrm{metab}}$ and $E_{\mathrm{mech}}$. $E_{\mathrm{lost}}$ relates to changes in RE that are not directly measurable, such as heat production, and are immeasurable and, as such, not taken into account by our model. Out of all these variables, conventional foot-worn sensors will always only reflect variation in $E_{\mathrm{ground}}$.

In Baumgartner et al., the authors show that foot-worn sensors that measure the acceleration at a single point do relate to the $\dot{V}O_{2}$ under steady conditions. Once the RE changes (or is forcibly altered, as in the above experiments), the relation between predicted running power (measured by a foot-worn sensor) and $\dot{V}O_{2}$ collapse. Beyond that, we show that the combination of sensors from the foot, chest, and lower arm contains even more information about the metabolic energy exertion of the athlete and, therefore, must carry implied information about the running economy of the athletes.

### 2.4. Extracting Information

In this work, we attempted to improve the measurement of $E_{\mathrm{mech}}$ by spreading more sensors over the body. This would yield a more representative measure of the forces and, thereby, the energy that is not directly applied to the ground but caused by additional movement. Modeling this “wasted” energy allows for a model that is overall more closely correlated with actual energy expenditure $\dot{V}O_{2}$, even under a modified running economy.

There is a large amount of variation in running form, and humans differ vastly when it comes to locomotion and the running economy [8,28,29]. Using data from just 29 athletes could never suffice to create a system that predicts the minute differences and variation over a larger population. To demonstrate this point, we performed a leave-one-out experiment, which managed to fit the training data but completely diverged from the validation data. The fact that our model did not perform on this task provides further evidence that it does not fit some unintended artifacts of our experimental setup.

In this work, we build a specific deep learning network, which we show to be incapable of fitting to random noise in our data. Next, we show that once we provide our actual data to the network, it is capable of performing regression between accelerometer A and $\dot{V}O_{2}$ data. Since it was not able to perform this regression on random noise, the model must have extracted and used signal (=information) about the $\dot{V}O_{2}$ from the acceleration data. We thereby show non-trivial mutual information between the $\dot{V}O_{2}$ and A. We additionally compare just a single acceleration sensor at the foot $A_{f}$ and three sensors on the foot, torso, and lower arm ${}^{t}A_{f}^{a}$, which in combination again contain more information about the $\dot{V}O_{2}$.

In the following, we first describe the network architecture used and how to transform the raw data from the accelerometer sensors into a single scalar prediction of the $\dot{V}O_{2}$. We then provide details about how we prove information content, given that the model has fewer trainable parameters than the input data.

#### 2.4.1. Deep Learning Model

The deep learning model for this study used data on the magnitude of accelerations at three body positions as the starting point. This data was transformed into the frequency domain using a fast Fourier transform (FFT), which allowed for the identification of patterns and trends in the data that may not have been immediately apparent in the time domain. Since the underlying movement is highly cyclical in nature, we are more interested in the separate frequencies of the motion and their magnitude rather than minor variations over time.

We designed the network by tuning it using foot-only data. We started with a full-capacity dense network, which can, of course, completely overfit and perfectly memorize and match this data. We then slimmed the model down while focusing on the prediction error of the foot-only data. Whenever a model change resulted in the model not being able to learn anything, we removed the last change.

The model utilizes small convolutional layers, which are a type of neural network layer that is particularly well-suited for analyzing and processing data that has a grid-like structure, such as images. In total, our final model had seven convolutional layers with ReLU activations for non-linearities and max pooling to reduce the dimensionality with every layer. The model consisted of a series of five 1D convolutional layers, each followed by a leaky ReLU non-linearity. Between each convolutional layer was a max pooling layer, which reduced the dimensionality of the data by taking the maximum value from a window of consecutive data points. The final layer was a dense layer with 10 units, followed by a final dense output layer with 1 unit, to perform the regression prediction. The model had a total of 228 trainable parameters θ, which was less than the 261 distinct data points it was trained on. Each layer, of course, contained even fewer parameters, and we tuned the network in a way to always have less than 29 parameters per layer. This meant that the model had fewer “knobs” or variables that could be adjusted during training, which made it unable to overfit.

Using this approach, we could, of course, have ended up with a large variety of different networks. More important than the exact architecture of the network is the fact that the model had a limited capacity. In the next section, we will show that our model could indeed determine whether or not the signal was present. To show that we, in fact, detected the proper signal and information about the underlying phenomenon, we showed that a lack of such a signal (i.e., just random input noise) could not have been trained by our network.

In every data, there was a certain signal-to-noise ratio. Without understanding the data, it was not possible to boost just the signal. Instead, we could vary the signal-to-noise ratio by drowning out the existing signal with additional artificial noise. We could be sure that the remaining signal in the modified data was very limited. If our model was able to perfectly fit such noisy data, it would mean that the model memorized some pattern in the noise to provide the correct prediction. We showed that our model did not learn anything from random noise. Next, we showed that the model did fit the original data (without added noise), which meant it had to encode the existing signal and, thereby, extract the mutual information between the input and output data.

#### 2.4.2. Indirect Proof of Mutual Information Content

In the following, we used concepts from information theory (according to Shannon [30]) to construct a proof using the information bottleneck. The term information (or signal/entropy) relates to the amount of surprise or variation contained in a variable before measurement. The mutual information between two variables, *X* and *Y*, is the amount of information that can be explained about *Y* after measuring *X* [31,32].

We attempted to show that there is information (i.e., a signal) contained in the distributed acceleration data. The amount of mutual information between $\dot{V}O_{2}$ and A is unknown. We showed that A must contain information about $\dot{V}O_{2}$ by fitting data to a model with a capacity that is lower than the degrees of freedom in our data. We used this mechanism of underfitting to show that the model must extract some commonalities about the data in order to predict the regression.

Imagine our model being a communication channel between the $\dot{V}O_{2}$ and A. In this metaphor, during backpropagation in training, our model tries to pass information from the $\dot{V}O_{2}$ to the A. If this channel is large enough, it could just learn a distinct pattern in the input A for each subject and test condition. Whenever this pattern is recognized, the model could put out the correct memorized $\dot{V}O_{2}$. Basically, a large channel would correspond to a large piece of paper on which the pattern for each data point could be written out on. As mentioned above, we limited the capacity of the model in a way to force it to learn something more about the data than just memorizing it. Through the low-capacity network, the metaphorical communication channel was reduced to a small piece of paper that could not even fit a description of each of the inputs. Since it was too small, the communicated information squeezed through our induced bottleneck needed to encode the data in a descriptive manner. It could only do so by writing out a recipe on how to convert the input to the output data. The existence of such a recipe implies that the input data contained mutual information with the output data.

As described before, we have a reasonable suspicion that the information about energy expenditure needs to be contained in the accelerometer data. As the subjects make more pronounced movements, they would spend more energy, as well as shown in an increase in the acceleration and vice versa. We now need to ask the question, how do we extract that information from the raw acceleration data? We want to do so in a way that prevents the network from memorizing anything about the data. We arrive at the above-described architecture, which chips away at the size of the data while never performing a layer transition that could carry enough information to identify a single input datum. The network can, therefore, not memorize the input data.

We manually varied the signal-to-noise ratio in the source data by adding additional noise. In terms of a communication channel, we clogged the channel with additional chatter, making it more difficult for the model to accurately transmit the information between the oxygen uptake $\dot{V}O_{2}$ measure to the acceleration A. By systematically varying the amount of noise in the source data, we could assess the robustness of the model and understand how it performed under different levels of signal-to-noise ratios. As we increased the signal/noise ratio, the model became incapable of fitting our data. This meant that the previous model (fitting data without noise) had learned to extract some signal about $\dot{V}O_{2}$ from A. It did not plainly memorize noise in the A data. This means that there is information about $\dot{V}O_{2}$ contained in A.

## 3. Results

In this section, we show the results of our experiment and how well the network with limited capacity was capable of fitting the respective input data. Since $\dot{V}O_{2}$ describes the *rate* of oxygen consumption, we took its average value over a certain time span. We normalize the $\dot{V}O_{2}$ for each athlete by their body weight and their current pace. For the Fourier transform, we sampled 128 frequencies. Thus, the overall dimensionalities of our data are $A:\left[\cdot ,128,3\right],\dot{V}O_{2}^{\mathrm{pred}}:\left[\cdot ,1\right]$. We keep the data dimension for noise, $A_{f}$, and ${}^{t}A_{f}^{a}$ the same to avoid artifacts from different formats. We transformed this input data step-wise through multiple layers, which reduced the dimensionality and added non-linearities (cf. Section 2.4.1).

Our experiments are based on the paradigm of how well an underpowered model can fit high-dimensional data. We considered data from 29 subjects, with nine conditions each: four during the initial stage test and five more with partially altered running economy. We got a total of 9×29=261 pairs of 60s segments of accelerometer data A and oxygen consumption $\dot{V}O_{2}$ for the same time frame. We trained the parameters θ of our model on partly overlapping snippets of 15 s to help bootstrap the data, which led to faster convergence using gradient descent. Our model trains a mapping from input A to output $\dot{V}O_{2}^{\mathrm{pred}}$:

(2) $$\begin{array}{c} \dot{V}O_{2}^{\mathrm{pred}}=f\left(A,\theta \right) \end{array}$$

where θ stands for the 228 trainable parameters of our model and f is the function, defined by our neural net architecture, that takes accelerometer data A and values for the parameters θ as the input to predict $\dot{V}O_{2}^{\mathrm{pred}}$. Now, to train the network, we found weights for the network parameters $\theta^{★}$ that minimized the difference between the prediction of $\dot{V}O_{2}^{\mathrm{pred}}$ and the actual measured $\dot{V}O_{2}$ using backpropagation via the Adam optimizer [33].

(3) $$\begin{array}{cc} L & =|\dot{V}O_{2}^{\mathrm{pred}}-\dot{V}O_{2}|\\ \theta^{★} & =\arg\min_{\theta }L \end{array}$$

**Figure 5:**
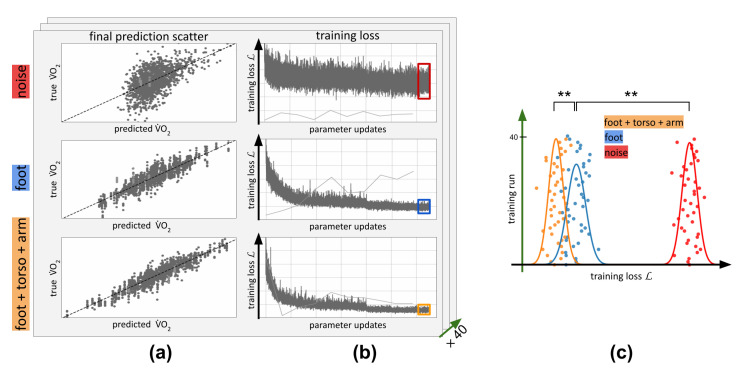
(**a**) Scatter plot of predicted $\dot{V}O_{2}$ (normalized by body weight and pace) versus correct $\dot{V}O_{2}$ for a completely trained network. Top: training on noise, middle: training on foot acceleration $A_{f}$, bottom: training on acceleration from the foot, torso, and arm ${}^{t}A_{f}^{a}$. The training target L is proportional to the coefficient of determination in this scatter plot and is optimized throughout training by updating the parameters of the model. (**b**) The progression of training loss L over time for our model in the three different conditions. The average loss L of the last 20 training steps (red/blue/orange boxes) is used to compare noise/foot/foot+torso+arm in (**c**). The single stray light gray shows the validation loss, which does not converge. (**c**) Averages of final training losses L over 40 repetitions. Orange: Foot + torso + arm, blue: foot, red: noise. Lines = fitted student-t distribution over the respective repetitions. **: statistically significant difference, *p* < 0.001.

We ran this optimization for 1000 epochs each; i.e., each data point was presented to the network 1000 times. In Figure 5, we show the training progression for different input data. Figure 5a, we depict scatter plots of the final prediction $\dot{V}O_{2}^{\mathrm{pred}}$ versus the actual $\dot{V}O_{2}$. Figure 5b shows how the training loss L decreased over time. In the first row of Figure 5a,b, we show the learning progression and prediction for training on random noise. It can be seen that our model basically did not learn anything at all, and the final prediction equals a random guess. The network did not manage to overfit. In the second row of Figure 5a,b, we show the same results for training on acceleration data from a single foot sensor $A_{f}$. Clearly, the data fit way better than when using random noise, and the training loss was properly minimized. In the final third row, we show an even better fit and lower training loss, which stemmed from an exemplary training run using acceleration data ${}^{t}A_{f}^{a}$ from three sensors at the foot, torso, and arm.

We repeated the training for 40 repetitions with different initial values for θ and data shuffling. In Figure 5c, we show the final training losses L for all training runs in a single plot, with the loss on the x-axis and the repeated training runs stacked on the y-axis. It can be seen that there were clear differences between the ability of the same architecture to minimize the loss when given noise as input (red), just accelerations from one foot (blue), or the combination of accelerations from the foot + torso + lower arm (three sensors, orange). There were big significant differences between just the foot and three sensors: p(foot + torso + arm, foot) = $3.23\times 10^{-18}$. There were also significant differences between foot and noise: p(foot, noise) = $6.90\;\times \;10^{-71}$. This proves that there is indeed mutual information about the oxygen consumption $\dot{V}O_{2}$ contained in measured acceleration data A.

## 4. Discussion

In this study, we aimed to investigate the connection between body-worn accelerometers and the metabolic cost of running. We demonstrated that there is a signal about the metabolic consumption $\dot{V}O_{2}$ contained in the acceleration at the foot $A_{f}$. Even more signal about the $\dot{V}O_{2}$ is contained in the combination of sensors at the foot, torso, and arm ${}^{t}A_{f}^{a}$. We proved this by fitting an underpowered neural network to experimental data and showed that it identifies and encodes signals about metabolic consumption.

We used an underpowered deep neural network and trained it to predict oxygen consumption $\dot{V}O_{2}$ from accelerometer data collected from three ${}^{t}A_{f}^{a}$ versus a single body position $A_{f}$. We demonstrated that the model is underpowered by showing that it is not capable of memorizing random noise of the same dimensionality as our training data. Additionally, the model had fewer trainable parameters than available data points. The results showed that the model was more successful at fitting the input when using accelerometer data from all three body positions ${}^{t}A_{f}^{a}$, as opposed to just one foot $A_{f}$ or noise. The differences in performance between these three conditions were found to be statistically significant.

We provided the neural network with more data points than trainable parameters θ. Therefore, in order to minimize the prediction loss L (cf. Equation (3)), the neural network had to encode the existing signal in A about $\dot{V}O_{2}$ in order to minimize this prediction error. We show that this same minimization was not possible for data with added noise. These results suggest that using accelerometer data from multiple body positions may be more precise for predicting $\dot{V}O_{2}$ when running compared to using data from just the foot.

This result seems very reasonable, considering the decomposition of various factors that combine into the metabolic cost (cf. Equation (1)). A foot-worn sensor only experiences forces $E_{\mathrm{ground}}$ that are proportional to the ground reaction force $F_{\mathrm{GRF}}$. Using three distributed sensors, we can additionally model the stabilizing motion and forces that are inherent to an athlete’s running form $E_{\mathrm{remain}}$, which all contribute to the running economy. By having a better measure for kinetic forces throughout the entire body with three sensors, we can more closely model the running economy and thereby potentially improve monitoring for long-term training progression.

Previous work has shown that using foot-worn sensors for assessing running power is only feasible in situations in which the running economy is not varied [11]. Our data exploration using just averages of magnitude in raw accelerometer data hints at this very same issue (cf. Figure 4). This means that these sensors are only useful for tracking changes in power output under a constant running economy but not for tracking changes in an athlete’s overall running performance over time [11]. When the running economy changes, as it naturally does with training and other factors, the relationship between metabolic cost and mechanical power becomes unreliable.

To remedy this shortcoming, we have shown a possibility to improve the predictive power of body-worn sensors for deriving an objective measure of running performance by predicting the metabolic cost of running $\dot{V}O_{2}$. We showed the mutual information between $\dot{V}O_{2}$ and body-worn accelerometers A. We also showed that three sensors contain significantly more information about the $\dot{V}O_{2}$ than just a single foot-worn sensor. We advocate for sport watch manufacturers to investigate combining data from sensors that many recreational runners already wear on their runs: foot power sensors, heart rate chest straps, and arm-worn watches.

### 4.1. Limitations

We encourage researchers with the respective companies to use sensors that are built into watches and heart rate monitors in addition to foot-worn sensors to better approximate energy consumption during running and thereby be stable to changes under varying running economies.

In Section 2.4, we mentioned that our collected data from 29 athletes, while valuable, will never be enough to model the vast differences in running forms for a wide population of runners. In fact, it might be an overall difficult task to ever collect enough data to achieve such a feat (if at all possible). We think that a different scale of data usage would be necessary to account for the complete range of variation. Solutions that use monocular human pose estimation [34], in combination with sports field registration [35] and kinetic approximation [36], seem like a feasible avenue.

While this study shows indications for improved measurements taking into account the running economy, it is limited in scope by being conducted in a lab setting on a treadmill. It has been shown that treadmill running can differ in energetics [37], as well as spatiotemporal parameters [38] from running overground. Further investigations should incorporate comparisons to running conditions in the field on varying terrains.

According to the convolutional theorem, convolutions in the frequency domain equate to multiplications in the time domain [39]. By first transforming our data into the frequency domain and then applying convolutional layers, we basically multiplied the raw accelerometer magnitude by different factors. As far as we are concerned, this does not have a direct interpretation of the information extraction process. We applied convolutional layers and max pooling to data from the frequency domain to be slightly more invariant to small shifts in the dominating frequencies in a subject’s running style. One could argue that the same could have been achieved by staying in the time domain and applying single multiplication steps with a final dense layer. For reasons of ease of implementation and having fewer parameters, we decided on the architecture described in Section 2.4.1. There is no clear interpretation of what it would mean to instead have stayed in the time domain and have applied convolutions there, so we leave explorations into this question to future work.

### 4.2. Future Work and Extensions

Our current approach used development kit sensor beacons (cf. Figure 3c). A more applied follow-up investigation should consider the actual sensors that recreational runners wear on their runs and use data streams from heart rate straps and watches. Scaling up the data collection beyond 29 athletes should provide a model that generalizes and can be used in practice. So far, we aimed to demonstrate that $\dot{V}O_{2}$ and A contain mutual information in order to motivate further larger-scale investigations. In order to build models that will generalize, different design choices should be made for the model itself. In this work, we switched from the time domain to the frequency domain in order to simplify the cyclical data and allow for models with very few parameters. Future iterations of this approach should consider model architectures that are specifically geared toward sequential data, such as transformer models [40].

## 5. Conclusions

This study showed that using accelerometer data from multiple body positions can improve the precision of predicting the metabolic cost of running, compared to using data from just a single foot. This has important implications for the use of body-worn sensors in tracking changes in running performance over time, as variations in the running economy can significantly impact the relationship between metabolic cost and mechanical power. To more accurately track changes in running performance, manufacturers of sports watches should consider incorporating data from multiple sensors, including those worn on the foot, chest, and wrist. We demonstrated that accelerometer data from sensors that are spread over the body in this way contain more information about the metabolic cost of running than commonly used foot-worn power meters. Since it has been shown that foot-worn sensors alone are not capable of adapting to changes in the running economy [11], such an improvement to wearable training documentation is necessary to adapt to long-term changes in athletes. Many recreational runners already wear the required sensors at our suggested locations. Therefore, the only thing missing in practice is to combine these streams of data. Doing so will open up possibilities of more detailed training prescriptions and faster progression toward optimal performance.

## Data Availability

Data and code implementation are available from the corresponding author upon reasonable request.
